# Supplementary material for: Applying Zinc Nutrient Reference Values as Proposed by Different Authorities Results in Large Differences in the Estimated Prevalence of Inadequate Zinc Intake by Young Children and Women and in Cameroon
Source: Nutrients. 2022 Feb 19;14(4):883. doi: 10.3390/nu14040883 (PMC8879783; doi:10.3390/nu14040883)
Supplement: Supplementary file 1 [file nutrients-14-00883-s001.zip › nutrients-1561738-supplementary.pdf]

## Supplementary files

**Table S1**: Summary of differences in total endogenous zinc losses and estimated fractional absorption of zinc between WHO, IOM, IZINCG and EFSA Zinc reference values

| Expert group | Endogenous zinc losses | | Fractional absorption |
|---|---|---|---|
| | Endogenous fecal zinc losses | Non-intestinal zinc losses | |
| WHO | • Estimated based on two balance (non-isotope tracer) studies, both with very low zinc intakes.<br>• Did not account for the fact that the EFZ is related to absorbed zinc, so should be estimated at the level of absorbed zinc that offsets all endogenous losses, EFZ was inflated by 40% to account for the reduced excretion of zinc with very restricted zinc intakes. | • The urinary zinc losses estimated by from balance (non-isotope tracer) studies were inflated by 40% to account for the reduced urinary excretion that occurs with very low zinc intakes.<br>• The integumentary zinc losses were estimated based on a single study of adult men and extrapolated to women<br>• WHO did not estimate zinc losses via semen or menstrual flow.<br>• The total non-intestinal zinc loss was estimated to be 0.60 mg/day. | • WHO established three dietary zinc reference values depending on the phytate zinc molar ratio. The diet types considered to represent a relatively high level of zinc absorption were 12 set of studies with no known source of zinc inhibitors. On the other hand, a combination of isotopically labeled single meals and whole day diets were used to represent a moderate and low zinc absorption levels. The level of zinc intake was not considered in these estimates.<br>• Assumed bioavailability, by diet type<br>  • High bioavailability: 50% FZA (phytate: Zn molar ratio < 5)<br>  • Moderate bioavailability: 30% FZA (phytate: Zn molar ratio 5 - 15)<br>  • Low bioavailability: 15% FZA (phytate: Zn molar ratio > 15) |
| IOM | • The EFZ was estimated based on radioactive or stable isotope tracer data generated from 10 whole-day diet studies conducted in North America and Europe [35].<br>• Linear regression was used to quantify the relationship between total absorbed zinc and EFZ. | • IOM estimated urinary zinc losses to be 0.63 mg/day, based on the average of 10 studies of men and women, with zinc intakes within the range in which urinary zinc excretion is constant.<br>• IOM estimated integumentary zinc losses based on a single study of adult males and extrapolated to women based on body surface area<br>• IOM assumed zinc losses via semen or menstrual flow is 0.1 mg/day. | • IOM used whole day diet mixed or semi-purified formula diet including zinc supplements consumed by men in North America and Europe to examine the relationship between the mean amount of absorbed zinc and total zinc ingested.<br>• IOM used a more conservative value of 30% as fractional absorption of zinc based on two studies conducted among infants and young children.<br>• The average fractional absorption of zinc for non-pregnant women was estimated to be 27% based on eight studies in which the average dietary intake was 10mg/day. |
| IZiNCG | • IZiNCG estimated EFZ losses based on 19 studies among men and women, irrespective of age and nationality, who consumed mixed diets based on | • IZINCG adopted the urinary and integumentary zinc losses estimates from IOM. | • IZiNCG used whole day diet studies of both men and women and excluded studies that used semi purified formula diets or zinc supplements when establishing the relationship between the total dietary zinc intake and the mean amount of absorbed zinc. |

| | | | |
|---|---|---|---|
| | common foods. IZiNCG excluded studies that used semi-purified formula diets and those using zinc supplements. The relationship between TAZ and EFZ losses were examined using linear regression for men and women separately and weighted by the sample size. | • IZINCG used the same data as IOM to estimate the integumentary losses but adjusted zinc losses in relation to body size for men and women rather than body surface area.<br>• IZINCG assumed estimated loss of zinc from menstrual flow to be negligible. | IZINCG classified diets as mixed or refined vegetarian diets or unrefined vegetarian diet based on phytate zinc molar ratio. IZiNCG assumed multiple fractional absorption levels (18%-34%) depending on the phytate:zinc molar ratio of the diet. |
| EFSA | • The EFSA estimated EFZ based on 10 stable isotope tracer studies (either compartmental modeling or fecal isotope dilution) that included 85 participants (31 males and 54 females) conducted United States, Europe, and China with dietary zinc intakes ranging from 0.8 to 29 mg/d from both conventional foods and semi-purified diets.<br>• EFSA used multiple regression analysis to examine the relationship between TAZ and EFZ. Body weight and EFZ were included as predictors of total absorbable zinc in the regression model. | • EFSA estimated integumental and sweat losses from men. The estimate for women was calculated by multiplying the value for men using female-to-male ratio of sweat zinc losses and whole-body sweat rates. The estimates for women was adjusted based on n by the female-to-male ratio of sweat zinc losses and whole-body sweat rates. | • EFSA used a trivariate response saturation model to examine the relationship between total zinc and phytate intakes and absorbed zinc. EFSA included only whole-day diet studies with a total of 72 points reflecting 650 individual measurements of total dietary zinc and phytate intake. |

**Table S2:** Predicted percentage point reduction of zinc inadequacy (± SE) among Cameroonian children following different levels of zinc fortification of bouillon cube in the presence of the existing wheat flour fortification program: estimated based on WHO, IOM, IZINCG, and EFSA dietary requirements and physiological requirement.

| Fortification levels | Macro-region | WHO | | IOM | | IZiNCG | | EFSA | |
|---|---|---|---|---|---|---|---|---|---|
| | | Physiological requirement, % ± SE | Dietary requirement, % ± SE | Physiological requirement, % ± SE | Dietary requirement, % ± SE | Physiological requirement, % ± SE | Dietary requirement, % ± SE | Physiological requirement, % ± SE | Dietary requirement, % ± SE |
| 0.6 mg/g | National | 4.2 ± 0.2 | 2.4 ± 0.4 | 4 ± 0.3 | 1.8 ± 0.3 | 0.9 ± 0.2 | 1.5 ± 0.3 | 6.8 ± 0.4 | 3.9 ± 0.3 |
| | South | 5.4 ± 0.3 | 3.1 ± 0.5 | 5.4 ± 0.4 | 2.4 ± 0.4 | 1.3 ± 0.3 | 2.1 ± 0.3 | 7.8 ± 0.5 | 5.1 ± 0.4 |
| | North | 3.9 ± 0.3 | 2.6 ± 0.3 | 3.7 ± 0.3 | 1.9 ± 0.4 | 0.9 ± 0.3 | 1.6 ± 0.3 | 6.3 ± 0.4 | 3.7 ± 0.4 |
| | Yaoundé/Douala | 1.9 ± 0.4 | 0.6 ± 0.2 | 1.0 ± 0.4 | 0.2 ± 0.2 | 0.1 ± 0.1 | 0.2 ± 0.1 | 5.3 ± 0.5 | 1.3 ± 0.4 |
| 1.2 mg/g | National | 8.3 ± 0.3 | 5.1 ± 0.6 | 7.7 ± 0.4 | 3.7 ± 0.6 | 1.5 ± 0.4 | 3.2 ± 0.5 | 15.4 ± 0.6 | 8.6 ± 0.6 |
| | South | 10.7 ± 0.5 | 6.4 ± 0.9 | 10.1 ± 0.7 | 4.9 ± 0.8 | 2.1 ± 0.5 | 4.2 ± 0.7 | 18.2 ± 1 | 10.9 ± 0.8 |
| | North | 7.9 ± 0.5 | 5.5 ± 0.6 | 7.6 ± 0.5 | 4.1 ± 0.6 | 1.5 ± 0.4 | 3.4 ± 0.5 | 14.2 ± 0.6 | 8.6 ± 0.6 |
| | Yaoundé/Douala | 3.4 ± 0.7 | 1.0 ± 0.4 | 1.7 ± 0.6 | 0.5 ± 0.2 | 0.1 ± 0.1 | 0.4 ± 0.2 | 10.6 ± 0.9 | 2.8 ± 0.7 |
| 1.8 mg/g | National | 8.9 ± 0.4 | 5.5 ± 0.7 | 8.2 ± 0.4 | 4.1 ± 0.6 | 1.6 ± 0.4 | 3.5 ± 0.6 | 16.8 ± 0.7 | 9.4 ± 0.6 |
| | South | 11.5 ± 0.6 | 7.0 ± 0.9 | 10.8 ± 0.7 | 5.3 ± 0.8 | 2.2 ± 0.5 | 4.6 ± 0.8 | 19.8 ± 1.1 | 11.7 ± 0.9 |
| | North | 8.5 ± 0.5 | 6.0 ± 0.6 | 8.2 ± 0.5 | 4.4 ± 0.6 | 1.6 ± 0.4 | 3.7 ± 0.6 | 15.6 ± 0.7 | 9.5 ± 0.6 |
| | Yaoundé/Douala | 3.5 ± 0.8 | 1.1 ± 0.4 | 1.8 ± 0.6 | 0.5 ± 0.3 | 0.2 ± 0.1 | 0.4 ± 0.2 | 11.3 ± 1 | 3.0 ± 0.8 |
| 3 mg/g | National | 11.5 ± 0.5 | 7.4 ± 0.8 | 10.4 ± 0.6 | 5.3 ± 0.8 | 2.0 ± 0.4 | 4.5 ± 0.7 | 23.3 ± 0.8 | 12.5 ± 0.8 |
| | South | 14.8 ± 0.7 | 9.1 ± 1.2 | 13.5 ± 0.9 | 6.8 ± 1.1 | 2.7 ± 0.6 | 5.7 ± 1.0 | 27.8 ± 1.3 | 15.5 ± 1.2 |
| | North | 11.2 ± 0.7 | 8.1 ± 0.8 | 10.6 ± 0.7 | 6.0 ± 0.8 | 2.0 ± 0.5 | 5.2 ± 0.7 | 22.1 ± 0.9 | 13.0 ± 0.8 |
| | Yaoundé/Douala | 4.1 ± 0.9 | 1.3 ± 0.3 | 2.1 ± 0.7 | 0.6 ± 0.3 | 0.2 ± 0.1 | 0.5 ± 0.3 | 14.5 ± 1.2 | 3.6 ± 0.9 |
| 5 mg/g | National | 14.1 ± 0.6 | 9.2 ± 1.0 | 12.3 ± 0.7 | 6.5 ± 0.9 | 2.4 ± 0.5 | 5.5 ± 0.8 | 30.1 ± 0.9 | 15.7 ± 1.0 |
| | South | 18.1 ±0.9 | 11.3 ± 1.4 | 15.9 ± 1.1 | 8.0 ± 1.3 | 3.2 ± 0.7 | 6.7 ± 1.2 | 35.8 ± 1.5 | 19.3 ± 1.6 |
| | North | 13.8 ± 0.8 | 10.2 ± 0.9 | 12.8 ± 0.8 | 7.5 ± 0.9 | 2.5 ± 0.5 | 6.4 ± 0.8 | 29.3 ± 1.1 | 16.7 ± 1.0 |
| | Yaoundé/Douala | 4.6 ± 1.0 | 1.5 ± 0.5 | 2.3 ± 0.7 | 0.7 ± 0.3 | 0.2 ± 0.1 | 0.6 ± 0.3 | 17.2 ± 1.3 | 4.2 ± 1.1 |

Absorbable zinc intake was used to estimate effective coverage based on physiological requirement. Total dietary zinc intake was used to estimate the prevalence of inadequate zinc intake based on dietary requirements. Based on phytate zinc molar ratio: the diet in Cameroon is classified as “moderately bioavailable”.

**Table S3:** Predicted percentage point reduction of zinc inadequacy (± SE) among Cameroonian children following wheat flour fortification and different levels of zinc fortification of bouillon cube in the absence of the existing wheat flour fortification program: estimated based on WHO, IOM, IZINCG, and EFSA dietary requirements and physiological requirement.

| | | WHO | | IOM | | IZiNCG | | EFSA | |
|---|---|---|---|---|---|---|---|---|---|
| Fortification levels | Macro-region | Physiological requirement, % ± SE | Dietary requirement, % ± SE | Physiological requirement, % ± SE | Dietary requirement, % ± SE | Physiological requirement, % ± SE | Dietary requirement, % ± SE | Physiological requirement, % ± SE | Dietary requirement, % ± SE |
| Bouillon Cube (0.6 mg/g) | National | 9.6 ± 0.4 | 5.7 ± 1.4 | 9.1 ± 0.5 | 4.0 ± 1.0 | 1.7 ± 0.6 | 3.2 ± 1.0 | 8.6 ± 0.5 | 11 ± 1.0 |
| | South | 11.1 ± 0.6 | 6.9 ± 1.8 | 10.6 ± 0.6 | 5.0 ± 1.1 | 2.3 ± 0.8 | 4.3 ± 1.2 | 8.7 ± 0.7 | 13.4 ± 1.3 |
| | North | 6.8 ± 0.4 | 3.8 ± 0.9 | 6.2 ± 0.4 | 2.3 ± 0.9 | 0.8 ± 0.4 | 1.8 ± 0.9 | 8.4 ± 0.5 | 7.4 ± 0.8 |
| | Yaoundé/Douala | 11.9 ± 1.0 | 7.0 ± 1.7 | 11.6 ± 1.0 | 4.9 ± 1.3 | 2.3 ± 0.8 | 3.8 ± 1.3 | 9.0 ± 0.6 | 13.5 ± 1.5 |
| Bouillon cube (1.2 mg/g) | National | 15.5 ± 0.6 | 9.4 ± 2.0 | 14.9 ± 0.7 | 6.7 ± 1.6 | 2.8 ± 0.9 | 5.3 ± 1.6 | 16.1 ± 0.9 | 18.2 ± 1.4 |
| | South | 18.3 ± 0.8 | 11.2 ± 2.6 | 17.6 ± 0.9 | 8.3 ± 1.7 | 3.7 ± 1.2 | 7.0 ± 1.8 | 16.7 ± 1.2 | 22.1 ± 1.8 |
| | North | 11.2 ± 0.6 | 6.5 ± 1.3 | 10.1 ± 0.6 | 4.2 ± 1.4 | 1.5 ± 0.6 | 3.3 ± 1.3 | 15.2 ± 0.7 | 12.5 ± 1.1 |
| | Yaoundé/Douala | 18.3 ± 1.2 | 11.1 ± 2.4 | 18.6 ± 1.5 | 7.6 ± 2.0 | 3.5 ± 1.1 | 6.0 ± 1.9 | 16.8 ± 1.1 | 21.6 ± 2.1 |
| Bouillon Cube (1.8 mg/g) | National | 19.5 ± 0.7 | 11.7 ± 2.4 | 18.9 ± 0.9 | 8.5 ± 1.9 | 3.5 ± 1.1 | 6.8 ± 1.9 | 22.6 ± 1.1 | 23.4 ± 1.7 |
| | South | 23.0 ± 0.9 | 14.1 ± 3.1 | 22.6 ± 1.1 | 10.5 ± 2.2 | 4.6 ± 1.5 | 8.8 ± 2.2 | 23.9 ± 1.5 | 28.4 ± 2.2 |
| | North | 14.1 ± 0.7 | 8.3 ± 1.6 | 13.1 ± 0.7 | 5.6 ± 1.7 | 2.0 ± 0.7 | 4.4 ± 1.6 | 20.7 ± 0.9 | 16.2 ± 1.3 |
| | Yaoundé/Douala | 22.7 ± 1.3 | 13.4 ± 2.8 | 22.5 ± 1.6 | 9.4 ± 2.3 | 4.1 ± 1.3 | 7.4 ± 2.2 | 23.6 ± 1.5 | 27.1 ± 2.5 |
| Bouillon Cube(3mg/g) | National | 24.7 ± 0.9 | 15.0 ± 2.8 | 23.9 ± 1.0 | 10.7 ± 2.3 | 4.3 ± 1.3 | 8.7 ± 2.2 | 32.7 ± 1.4 | 29.9 ± 1.9 |
| | South | 29.3 ± 1.2 | 17.9 ± 3.6 | 28.5 ± 1.3 | 13.3 ± 2.6 | 5.6 ± 1.7 | 11.1 ± 2.6 | 35.5 ± 2.0 | 36.1 ± 2.5 |
| | North | 17.9 ± 0.8 | 10.9 ± 1.9 | 16.9 ± 0.9 | 7.4 ± 2.0 | 2.8 ± 0.9 | 6.1 ± 1.8 | 28.8 ± 1.1 | 21.3 ± 1.6 |
| | Yaoundé/Douala | 28.3 ± 1.6 | 16.5 ± 3.3 | 27.4 ± 1.8 | 11.3 ± 2.7 | 4.8 ± 1.4 | 9.0 ± 2.5 | 34.1 ± 2.0 | 33.5 ± 2.7 |
| Bouillon Cube(5mg/g) | National | 29.5 ± 1.0 | 18.1 ± 3.1 | 28.0 ± 1.0 | 12.8 ± 2.5 | 5.0 ± 1.4 | 10.3 ± 2.4 | 44.0 ± 1.6 | 35.7 ± 2.0 |
| | South | 34.9 ± 1.3 | 21.5 ± 4.0 | 33.4 ± 1.3 | 15.6 ± 2.9 | 6.5 ± 1.8 | 12.8 ± 2.9 | 48.5 ± 2.3 | 42.7 ± 2.6 |
| | North | 21.7 ± 1.0 | 13.8 ± 2.2 | 20.4 ± 0.9 | 9.5 ± 2.2 | 3.3 ± 1.0 | 7.8 ± 2.0 | 38.3 ± 1.3 | 26.6 ± 1.7 |
| | Yaoundé/Douala | 32.9 ± 1.7 | 18.8 ± 3.6 | 31.2 ± 1.9 | 12.7 ± 2.9 | 5.3 ± 1.5 | 10.0 ± 2.7 | 45.2 ± 2.2 | 38.5 ± 2.9 |
| | National | 18.7 ± 0.8 | 10.3 ± 2.8 | 17.7 ± 0.9 | 7.0 ± 2.3 | 2.7 ± 1.2 | 5.4 ± 2.2 | 24.4 ± 1.1 | 23.0 ± 1.5 |

| | | | | | | | | | |
|---|---|---|---|---|---|---|---|---|---|
| Wheat flour (95 mg/kg) | South | 20.3 ± 1.3 | 11.9 ± 3.6 | 19.7 ± 1.4 | 8.5 ± 2.5 | 3.4 ± 1.5 | 6.9 ± 2.5 | 23.1 ± 1.5 | 26.9 ± 2.1 |
| | North | 9.2 ± 0.9 | 4.3 ± 2 | 8.5 ± 0.9 | 2.3 ± 2 | 0.5 ± 0.9 | 1.6 ± 1.9 | 15.3 ± 1.1 | 11.6 ± 1.3 |
| | Yaoundé/Douala | 35.6 ± 1.5 | 19.3 ± 3.5 | 33 ± 1.8 | 13 ± 2.8 | 5.9 ± 1.5 | 10.2 ± 2.7 | 48.3 ± 2.3 | 39.1 ± 2.1 |

Absorbable zinc intake was used to estimate effective coverage based on physiological requirement. Total dietary zinc intake was used to estimate the prevalence of inadequate zinc intake based on dietary requirements. Based on phytate zinc molar ratio: the diet in Cameroon is classified as “moderately bioavailable”
.

**Table S4*:* :** Predicted percentage point reduction of zinc inadequacy (± SE) among Cameroonian women following different levels of zinc fortification of bouillon cube in the presence of the existing wheat flour fortification program: estimated based on WHO, IOM, IZINCG, and EFSA physiological and dietary requirements.

| Bouillon cube Fortification levels | Macro-region | **WHO** | | **IOM** | | **IZINCG** | | **EFSA** | | Corrected IOM and IZiNCG Values[1] | |
|---|---|---|---|---|---|---|---|---|---|---|---|
| | | Physiological requirement, % ± SE | Dietary requirement, % ± SE | Physiological requirement, % ± SE | Dietary requirement, % ± SE | Physiological requirement, % ± SE | Dietary requirement, % ± SE | Physiological requirement, % ± SE | Dietary requirement, % ± SE | IOM, % ± SE | IZINCG, % ± SE |
| 0.6 mg/g | National | 1 ± 0.2 | 1.3 ± 0.3 | 4.3 ± 0.2 | 6.5 ± 0.3 | 5.3 ± 0.2 | 5.7 ± 0.3 | 8 ± 0.3 | 8.1 ± 0.3 | 7.5 ± 0.3 | 7.9 ± 0.3 |
| | South | 2 ± 0.3 | 2.8 ± 0.4 | 1.3 ± 0.2 | 10.7 ± 0.6 | 8.4 ± 0.4 | 9.9 ± 0.6 | 8.6 ± 0.5 | 11.8 ± 0.8 | 8 ± 0.5 | 8.8 ± 0.5 |
| | North | 0.3 ± 0.1 | 0.1 ± 0.1 | 7.2 ± 0.4 | 2.5 ± 0.5 | 3.2 ± 0.3 | 2 ± 0.4 | 7.2 ± 0.2 | 4.2 ± 0.5 | 6.7 ± 0.3 | 6.8 ± 0.3 |
| | Yaoundé /Douala | 0.1 ± 0.1 | 0.1 ± 0.1 | 6.4 ± 0.5 | 3.5 ± 0.5 | 1.9 ± 0.2 | 2.6 ± 0.5 | 7.8 ± 0.4 | 6.2 ± 0.5 | 7.5 ± 0.4 | 7.7 ± 0.4 |
| 1.2 mg/g | National | 1.3 ± 0.2 | 2 ± 0.4 | 9.7 ± 0.4 | 11.1 ± 0.5 | 8.8 ± 0.4 | 9.6 ± 0.6 | 14.9 ± 0.5 | 14.5 ± 0.6 | 14 ± 0.4 | 14.7 ± 0.4 |
| | South | 2.8 ± 0.5 | 4.2 ± 0.7 | 3.5 ± 0.6 | 18.9 ± 1 | 14.1 ± 0.7 | 16.9 ± 1 | 16.9 ± 0.8 | 22.1 ± 1.3 | 15.7 ± 0.7 | 17 ± 0.7 |
| | North | 0.3 ± 0.1 | 0.2 ± 0.1 | 15.2 ± 1 | 3.9 ± 0.8 | 5.2 ± 0.5 | 3.2 ± 0.6 | 13.2 ± 0.4 | 6.8 ± 1 | 12.2 ± 0.6 | 12.7 ± 0.4 |
| | Yaoundé /Douala | 0.1 ± 0.1 | 0.2 ± 0.1 | 14.4 ± 0.9 | 5.3 ± 0.8 | 2.8 ± 0.4 | 4 ± 0.7 | 13.3 ± 0.7 | 9.9 ± 1 | 12.8 ± 0.6 | 12.7 ± 0.6 |
| 1.8 mg/g | National | 1.5 ± 0.3 | 2.4 ± 0.5 | 15.3 ± 0.6 | 14.4 ± 0.6 | 11.1 ± 0.5 | 12.3 ± 0.7 | 20.7 ± 0.6 | 19.4 ± 0.8 | 19.4 ± 0.6 | 20.5 ± 0.6 |
| | South | 3.2 ± 0.5 | 5.1 ± 0.8 | 6.8 ± 0.8 | 25 ± 1.3 | 18.1 ± 0.9 | 21.9 ± 1.2 | 24.1 ± 0.9 | 30.7 ± 1.8 | 22.5 ± 0.9 | 24.4 ± 0.9 |
| | North | 0.4 ± 0.2 | 0.2 ± 0.1 | 22.8 ± 1.3 | 4.7 ± 0.9 | 6.5 ± 0.7 | 3.9 ± 0.8 | 18.3 ± 0.6 | 8.3 ± 1.2 | 17 ± 0.7 | 17.9 ± 0.5 |
| | Yaoundé /Douala | 0.1 ± 0.1 | 0.2 ± 0.2 | 22.2 ± 1.3 | 6.4 ± 1 | 3.2 ± 0.5 | 4.8 ± 0.9 | 16.9 ± 0.8 | 12.1 ± 1.3 | 16.4 ± 0.8 | 16.3 ± 0.8 |
| 3 mg/g | National | 1.7 ± 0.3 | 2.7 ± 0.5 | 27.1 ± 1 | 18.3 ± 0.9 | 13.8 ± 0.7 | 15.2 ± 1 | 29.4 ± 0.8 | 25.5 ± 1 | 27.5 ± 0.7 | 29.3 ± 0.7 |
| | South | 3.6 ± 0.6 | 5.7 ± 1 | 16.1 ± 1.2 | 32.5 ± 1.6 | 22.6 ± 1.2 | 27.3 ± 1.6 | 35.6 ± 1.2 | 42 ± 2.2 | 32.8 ± 1.2 | 35.9 ± 1.2 |
| | North | 0.4 ± 0.2 | 0.2 ± 0.1 | 36.1 ± 2 | 5.6 ± 1.1 | 8.0 ± 0.9 | 4.6 ± 1 | 25.7 ± 0.8 | 9.8 ± 1.5 | 24 ± 0.9 | 25.5 ± 0.7 |
| | Yaoundé /Douala | 0.1 ± 0.1 | 0.2 ± 0.2 | 36.9 ± 2.1 | 7.3 ± 1.2 | 3.6 ± 0.6 | 5.5 ± 1 | 21.5 ± 1.1 | 14 ± 1.7 | 21.1 ± 1 | 20.9 ± 1 |
| 5 mg/g | National | 1.9 ± 0.4 | 2.8 ± 0.6 | 44 ± 1.7 | 21.1 ± 1.1 | 15.8 ± 1.5 | 17.2 ± 1.1 | 38.3 ± 2.4 | 30.3 ± 1.1 | 35.9 ± 1.2 | 38.5 ± 3.3 |
| | South | 3.9 ± 0.7 | 6 ± 1.1 | 34.6 ± 1.6 | 38.1 ± 1.8 | 26.3 ± 2.2 | 31.2 ± 1.9 | 48 ± 2.1 | 51.2 ± 2.3 | 43.8 ± 1.5 | 48.7 ± 2.7 |

| | | | | | | | | | | | |
|---|---|---|---|---|---|---|---|---|---|---|---|
| | North | 0.4 ± 0.2 | 0.2 ± 0.1 | 51.2 ± 2.6 | 6.2 ± 1.3 | 9.0 ± 1.7 | 5 ± 1.2 | 33.2 ± 3.8 | 11 ± 1.8 | 31.4 ± 1.3 | 33.2 ± 5.5 |
| | Yaoundé /Douala | 0.1 ± 0.1 | 0.2 ± 0.2 | 53.2 ± 2.8 | 7.7 ± 1.4 | 3.8 ± 0.6 | 5.9 ± 1.2 | 25.3 ± 2.4 | 15 ± 1.9 | 25.4 ± 2.7 | 24.6 ± 2.1 |

Absorbable zinc intake was used to estimate effective coverage based on physiological requirement. Total dietary zinc intake was used to estimate the prevalence of inadequate zinc intake based on dietary requirements. Based on phytate zinc molar ratio: the diet in Cameroon is classified as “moderately bioavailable”.

[1]Hambidge et al. (2011) re-calculated the estimated physiological zinc requirement for adults proposed by IOM and IZiNCG [1]

**Table S5:** Predicted percentage point reduction of zinc inadequacy (± SE) among Cameroonian women following wheat flour fortification and different levels of zinc fortifification of bouillon cube in the absence of the existing wheat flour fortification program: estimated based on WHO, IOM, IZINCG, and EFSA physiological and dietary requirements.

| Fortification levels | Macro-region | WHO | | IOM | | IZINCG | | EFSA | | Corrected IOM and IZiNCG Values[1] | |
|---|---|---|---|---|---|---|---|---|---|---|---|
| | | Physiological requirement, % ± SE | Dietary requirement, % ± SE | Physiological requirement, % ± SE | Dietary requirement, % ± SE | Physiological requirement, % ± SE | Dietary requirement, % ± SE | Physiological requirement, % ± SE | Dietary requirement, % ± SE | IOM, % ± SE | IZINCG, % ± SE |
| Bouillon Cube (0.6 mg/g) | National | 1.7 ± 0.3 | 4.3 ± 0.4 | 5.7 ± 0.3 | 13.3 ± 0.7 | 10.4 ± 0.3 | 14.5 ± 0.8 | 9.3 ± 0.3 | 11.4 ± 0.4 | 8.6 ± 0.4 | 9.6 ± 0.4 |
| | South | 3.1 ± 0.4 | 7.2 ± 0.7 | 2.7 ± 0.4 | 16.2 ± 1.2 | 13.3 ± 0.6 | 19.1 ± 1.2 | 6.1 ± 0.6 | 10.9 ± 0.9 | 5.5 ± 0.6 | 6.7 ± 0.7 |
| | North | 0.1 ± 0.1 | 0.1 ± 0.1 | 9.2 ± 0.6 | 5.1 ± 0.6 | 5.4 ± 0.4 | 3.9 ± 1.0 | 11.3 ± 0.5 | 9.1 ± 0.9 | 10.9 ± 0.6 | 10.6 ± 0.6 |
| | Yaoundé /Douala | 1.0 ± 0.3 | 4.5 ± 0.6 | 6.7 ± 0.6 | 19.6 ± 1.4 | 11.9 ± 0.5 | 20.8 ± 1.1 | 13.2 ± 0.7 | 16 ± 1.1 | 12.2 ± 0.7 | 14.3 ± 0.7 |
| Bouillon Cube (1.2 mg/g) | National | 2.2 ± 0.4 | 5.9 ± 0.7 | 13.5 ± 0.6 | 24.4 ± 1.3 | 17.3 ± 0.6 | 25 ± 0.7 | 19.8 ± 0.7 | 23.5 ± 1 | 18.5 ± 0.7 | 20 ± 0.7 |
| | South | 4.0 ± 0.7 | 10.1 ± 1.1 | 8.1 ± 0.9 | 32.1 ± 2.3 | 23.4 ± 0.9 | 34.5 ± 1.2 | 15.5 ± 1.2 | 25.8 ± 1.8 | 14.1 ± 1.1 | 16.6 ± 1.2 |
| | North | 0.1 ± 0.1 | 0.2 ± 0.1 | 18.2 ± 1 | 7.7 ± 1.1 | 8.4 ± 0.7 | 6 ± 0.9 | 20.3 ± 0.8 | 13.8 ± 1.3 | 19.7 ± 0.9 | 19.1 ± 0.7 |
| | Yaoundé /Douala | 1.2 ± 0.3 | 5.7 ± 1 | 18.1 ± 1.1 | 33.6 ± 2.1 | 18.0 ± 0.7 | 33.8 ± 0.9 | 28 ± 1.2 | 33.7 ± 2.4 | 26.5 ± 1.2 | 29 ± 1.3 |
| Bouillon Cube (1.8 mg/g) | National | 2.4 ± 0.4 | 6.6 ± 0.9 | 21.8 ± 0.7 | 32.2 ± 1.6 | 21.8 ± 0.7 | 31.3 ± 0.9 | 29 ± 0.9 | 34.3 ± 1.7 | 27.4 ± 0.9 | 29.1 ± 0.9 |
| | South | 4.3 ± 0.8 | 11.3 ± 1.4 | 15.3 ± 1.3 | 44 ± 2.8 | 30.3 ± 1.2 | 44.3 ± 1.4 | 25 ± 1.5 | 40.6 ± 2.8 | 23.1 ± 1.4 | 26.1 ± 1.5 |
| | North | 0.1 ± 0.1 | 0.2 ± 0.1 | 26 ± 1.2 | 9.1 ± 1.3 | 10.1 ± 0.9 | 7 ± 1.2 | 27.4 ± 1 | 16.5 ± 1.7 | 26.4 ± 1 | 25.8 ± 0.9 |
| | Yaoundé /Douala | 1.3 ± 0.4 | 6.1 ± 1.1 | 29.5 ± 1.3 | 42.4 ± 2.4 | 21.7 ± 0.9 | 40.8 ± 1.1 | 40.1 ± 1.5 | 48.4 ± 3.2 | 38.4 ± 1.5 | 40.7 ± 1.5 |
| Bouillon Cube (3mg/g) | National | 2.5 ± 0.5 | 6.9 ± 1 | 35.6 ± 0.9 | 40.8 ± 1.6 | 26.6 ± 0.9 | 37.3 ± 1.0 | 42.9 ± 1.2 | 48.4 ± 2.4 | 40.3 ± 1.1 | 42.9 ± 1.1 |
| | South | 4.6 ± 0.8 | 11.8 ± 1.6 | 29.5 ± 1.6 | 58 ± 3.1 | 38.1 ± 1.4 | 54 ± 1.6 | 41.1 ± 1.7 | 62.3 ± 4 | 38.1 ± 1.7 | 42 ± 1.8 |
| | North | 0.1 ± 0.1 | 0.2 ± 0.1 | 37 ± 1.3 | 10.2 ± 1.6 | 11.8 ± 1.2 | 7.9 ± 1.3 | 37.4 ± 1.2 | 18.9 ± 2 | 35.6 ± 1.2 | 35.9 ± 1.1 |

| | | | | | | | | | | | |
|---|---|---|---|---|---|---|---|---|---|---|---|
| | Yaoundé/Douala | 1.3 ± 0.4 | 6.3 ± 1.2 | 46.8 ± 1.4 | 50.6 ± 2.3 | 24.6 ± 1.1 | 46.5 ± 1.1 | 55.2 ± 1.7 | 64.1 ± 3.6 | 52.7 ± 1.6 | 55.6 ± 1.7 |
| Bouillon Cube (5mg/g) | National | 2.5 ± 0.5 | 7 ± 1 | 50.2 ± 1.2 | 45.5 ± 1.2 | 29.4 ± 1.0 | 40.4 ± 0.3 | 56.4 ± 1.4 | 57.4 ± 2 | 52.7 ± 1.2 | 56.6 ± 1.5 |
| | South | 4.7 ± 0.8 | 12.1 ± 1.7 | 46.6 ± 1.8 | 66.3 ± 2.3 | 42.9 ± 1.6 | 59.3 ± 0.6 | 57.8 ± 1.9 | 77.4 ± 3.6 | 53.1 ± 1.7 | 59 ± 2.1 |
| | North | 0.1 ± 0.2 | 0.2 ± 0.1 | 48.1 ± 1.4 | 10.8 ± 1.8 | 12.9 ± 1.3 | 8.3 ± 0.4 | 47.3 ± 1.5 | 20.1 ± 2.3 | 44.7 ± 1.4 | 46.2 ± 1.5 |
| | Yaoundé /Douala | 1.3 ± 0.4 | 6.3 ± 1.2 | 61.3 ± 1.5 | 54 ± 2.2 | 25.8 ± 1.1 | 49.1 ± 0.5 | 67.3 ± 1.6 | 71.7 ± 2.8 | 64.1 ± 1.5 | 67.5 ± 1.7 |
| Wheat flour (95 mg/kg) | National | 0.5 ± 0.4 | 4.1 ± 0.9 | 20.1 ± 0.8 | 24.5 ± 1.2 | 13.6 ± 0.8 | 23.3 ± 1.7 | 24.3 ± 0.8 | 27.8 ± 1.3 | 23.0 ± 0.8 | 24.4 ± 0.8 |
| | South | 0.6 ± 0.8 | 6.0 ± 1.5 | 10.8 ± 0.9 | 28.3 ± 1.9 | 16.6 ± 1.3 | 28.1 ± 1.5 | 16.9 ± 1.2 | 27.0 ± 1.9 | 15.6 ± 1.2 | 17.6 ± 1.2 |
| | North | 0.0 ± 0.2 | 0 ± 0.1 | 18.0 ± 1.4 | 4.5 ± 1.3 | 3.7 ± 0.9 | 3.2 ± 1.9 | 17.3 ± 1.2 | 9.0 ± 2.0 | 16.9 ± 1.2 | 16.4 ± 1.1 |
| | Yaoundé /Douala | 1.2 ± 0.4 | 6.1 ± 1.2 | 43.5 ± 1.9 | 47.0 ± 2.3 | 22.4 ± 1.1 | 43.8 ± 2.4 | 51.1 ± 2.0 | 58.6 ± 3.1 | 48.9 ± 1.9 | 51.6 ± 2.0 |

Absorbable zinc intake was used to estimate effective coverage based on physiological requirement. Total dietary zinc intake was used to estimate the prevalence of inadequate zinc intake based on dietary requirements. Based on phytate zinc molar ratio: the diet in Cameroon is classified as “moderately bioavailable”.

[1]Hambidge et al. (2011) re-calculated the estimated physiological zinc requirement for adults proposed by IOM and IZiNCG [1]

**Table S6:** Prevalence (± SE) of zinc intake above the UL considering the contribution of zinc fortification programs among children and women in Cameroon by macro region: estimated based on WHO, IOM, IZINCG, and EFSA UL reference values.

| | | Children | | | | Women | | |
|---|---|---|---|---|---|---|---|---|
| Fortification levels | Macro regions | WHO | IOM | IZINCG | EFSA | WHO | IOM /IZINCG | EFSA |
| Baseline (no fortification) | South | 6.7 ± 1.5 | 7.1 ± 1.5 | 6.8 ± 1.5 | 7.1 ± 1.5 | 0 ± 0 | 0 ± 0 | 0.1 ± 0 |
| | North | 1.0 ± 0.2 | 9.5 ± 2.1 | 4.3 ± 1.6 | 9.6 ± 2.2 | 0 ± 0 | 0 ± 0 | 0.3 ± 0 |
| | Yaoundé /Douala | 5.0 ± 0.5 | 6.0 ± 1.1 | 5.2 ± 0.6 | 6.0 ± 1.1 | 0 ± 0 | 0 ± 0 | 0 ± 0. |
| Wheat flour (95 mg/kg) | South | 6.8 ± 1.5 | 27.7 ± 2 | 20.5 ± 1.9 | 29.0 ± 2.1 | 0 ± 0 | 0 ± 0 | 0.2 ± 0.1 |
| | North | 1.1 ± 0.2 | 29.3 ± 1.8 | 21.7 ± 1.6 | 30.9 ± 1.8 | 0.3 ± 0.2 | 0.1 ± 0.1 | 6.0 ± 1.3 |
| | Yaoundé /Douala | 5.5 ± 0.6 | 55.2 ± 2 | 45.4 ± 1.8 | 57.4 ± 2.1 | 0.1 ± 0.1 | 0 ± 0 | 2.2 ± 0.7 |
| Bouillon cube (5 mg/g) | South | 7.2 ± 1.5 | 54.4 ± 2.5 | 45.2 ± 2 | 56.5 ± 2.6 | 0.7 ± 0.4 | 0.2 ± 0.2 | 7.2 ± 1.7 |
| | North | 2.9 ± 0.6 | 56 ± 2.1 | 47.7 ± 1.8 | 58.2 ± 2.3 | 8.7 ± 2.2 | 3.3 ± 1.3 | 41.4 ± 1.9 |
| | Yaoundé /Douala | 6.1 ± 0.7 | 57.3 ± 2.5 | 48.5 ± 2.0 | 59.3 ± 2.7 | 1.0 ± 0.6 | 0.2 ± 0.2 | 12.0 ± 2.4 |
| Bouillon cube (5 mg/g) + Wheat flour (95 mg/kg) | South | 9.6 ± 1.7 | 68.2 ± 2.5 | 59.8 ± 2.4 | 70.1 ± 2.6 | 3.1 ± 0.8 | 1.3 ± 0.4 | 16.7 ± 1.7 |
| | North | 6.0 ± 0.7 | 63.1 ± 2.2 | 55.8 ± 2.0 | 65.0 ± 2.2 | 19.1 ± 1.8 | 9.8 ± 1.6 | 53.0± 2.3 |
| | Yaoundé /Douala | 16.5 ± 1.3 | 81.0 ± 1.9 | 76.1 ± 2.1 | 82.0 ± 1.9 | 12.8 ± 1.8 | 6.0± 1.3 | 43.6 ± 2 |

Reference

1. Hambidge, K.M.; Miller, L.V.; Krebs, N.F. Physiological requirements for zinc. *Int. J. Vitam. Nutr. Res.* **2011**, *81*, 72–78. https://doi.org/10.1024/0300-9831/a00052.
